# The Role of Spatial Configuration in Multiple Identity Tracking

**DOI:** 10.1371/journal.pone.0093835

**Published:** 2014-04-09

**Authors:** Lei Zhao, Qiyang Gao, Yan Ye, Jifan Zhou, Rende Shui, Mowei Shen

**Affiliations:** Department of Psychology and Behavioral Sciences, Zhejiang University, Hangzhou, China; Centre de Neuroscience Cognitive, France

## Abstract

**Background:**

The simultaneous tracking and identification of multiple moving objects encountered in everyday life requires one to correctly bind identities to objects. In the present study, we investigated the role of spatial configuration made by multiple targets when observers are asked to track multiple moving objects with distinct identities.

**Methodology/Principal Findings:**

The overall spatial configuration made by the targets was manipulated: In the constant condition, the configuration remained as a virtual convex polygon throughout the tracking, and in the collapsed condition, one of the moving targets (critical target) crossed over an edge of the virtual polygon during tracking, destroying it. Identification performance was higher when the configuration remained intact than when it collapsed (Experiments 1a, 1b, and 2). Moreover, destroying the configuration affected the allocation of dynamic attention: the critical target captured more attention than did the other targets. However, observers were worse at identifying the critical target and were more likely to confuse it with the targets that formed the virtual crossed edge (Experiments 3–5). Experiment 6 further showed that the visual system constructs an overall configuration only by using the targets (and not the distractors); identification performance was not affected by whether the distractor violated the spatial configuration.

**Conclusions/Significance:**

In sum, these results suggest that the visual system may integrate targets (but not distractors) into a spatial configuration during multiple identity tracking, which affects the distribution of dynamic attention and the updating of identity-location binding.

## Introduction

The visual system is challenged when one is required to track multiple moving objects in a dynamic visual environment. For example, when playing basketball, one must simultaneously keep track of numerous teammates who are constantly changing positions. Thus, awareness of “who” and “where” are both important factors when making quick yet rational decisions, such as passing the ball to the correct player. This type of task involves dynamic binding of identity and spatiotemporal information [1]. To complete the task, we must continuously bind multiple identities to the correct objects while they are moving and changing locations.

Pylyshyn and Storm (1988) were the first to investigate an observer’s ability to track multiple moving objects [2]. Their multiple-object tracking (MOT) paradigm requires observers to simultaneously track a subset of moving targets presented on a computer monitor among a group of identical distractors. Investigations conducted by Pylyshyn and Storm among others, have shown that observers can simultaneously track four or five targets among identical distractors [3]–[7]. A majority of this research has focused on the spatiotemporal factors that influence stimulus tracking, and has revealed that the speed, motion information and density of moving objects plays an important role [8]–[13]. However, relatively little research has focused on targets with unique identities.

The few studies that have investigated the role of identity in MOT focused on the observer’s ability to maintain identity-location bindings during the tracking procedure; their results indicated that observers were better at tracking objects than they were at identifying them. For example, when each target was assigned an identity at the beginning of the tracking task, the observers were unable to remember those identities even when they correctly tracked the targets [14]. There is also evidence that requiring observers to report features (e.g., color, shape) of disoccluded or reappeared objects does not result in differential performance for tracked and untracked objects. This suggests that features of the object may not be encoded even when the observer attended to the object [15]. Other studies, however, have shown that featural information is accessible during tracking, and that observers can hold approximately two targets in this type of task. These results indicate that there may be two separate systems involved in multiple identity tracking: one for positional, and one for featural information [16], [17].

Some studies investigating dynamic attention have suggested that there is a serial process operating during multiple identity tracking such that each target’s identity-location binding must be refreshed sequentially [18]–[20]. In light of these findings, a serial switching model of multiple identity tracking (MOMIT) has been proposed [19]. In the model, identity-location bindings are held in the episodic buffer with a capacity limitation, and the efficient updating of these bindings is assumed to be a non-automatic process based on continuous shifts of attention between targets. As targets move continuously, a location error may occur in the updating process, which causes a failure of binding. However, the overall representation of these multiple bindings and how the switch is made remains unknown. In the present study, we investigated the organization and maintenance of multiple identities when multiple objects are tracked. In particular, we examined the overall representation of multiple identity-location bindings (i.e., how featural properties and spatiotemporal information are integrated during tracking), and whether spatial configuration plays a critical role in this process.

There is substantial empirical evidence to suggest that a higher-order structure and prior knowledge (e.g., perceptual organization) are utilized when visual objects are encoded or even memorized [21]–[23]. For instance, people encode a real-world scene into a perceptual and conceptual gist plus detailed information, and use this gist to guide their choices [24], [25]. Grouping objects together into perceptual units results in better visual working memory performance [26], [27]. This suggests that tracking multiple unique targets may be influenced by the perceptual organization of the identity-location bindings. However, the visual system’s representation and organization of these bindings have not been fully determined. As a result, it is largely unknown whether spatial configuration affects the identification process, because multiple identity-location bindings may also be integrated into perceptual units by spatial configuration.

Spatial configuration has been actively investigated in static scenes [26], [28]–[31]. For example, in a study using the change-detection task [32], displays that included four items with featural properties (colors or shapes) located in four different quadrants were presented to the participants in both the memory and probe images. Participants were asked to decide whether the featural properties in the corresponding quadrant had changed. The appearance of the probe images was manipulated in three ways so as to vary the location or configuration of the four items. Results showed that performance was not affected by location changes of individual items, as long as the global configuration of all the items was preserved. This suggests the representation of identity-location bindings is related to spatial configuration rather than the respective items per se.

Other studies using the standard MOT paradigm have shown that perceptual grouping may play an important role in successful tracking [13], [33]. For instance, Yantis (1992) showed that manipulating the initial formation and maintenance of a perceptual grouping when the targets were in motion affected tracking performance [13]. The initial positions were manipulated by either selecting them at random or as the vertices of a canonical polygon (a regular triangle, diamond, or pentagon); tracking performance was significantly better in the canonical polygon condition (Experiment 1). Yantis also manipulated the targets’ spatial configuration in the canonical polygon condition during tracking (Experiment 4). When the configuration was randomly determined and yielded frequent object collapses (the unconstrained condition), tracking performance was worse than when the configuration was constrained to a non-rigid convex polygon (the constrained condition). Yantis concluded that observers spontaneously grouped the targets and directed their attention toward this coherent but non-rigid virtual object during tracking.

The MOT paradigm has also been utilized in several eye-tracking studies, which suggest a center-looking strategy during MOT. In other words, observers tend to look at the center of the virtual polygon formed by the targets [34]–[36]. This center-looking strategy facilitates tracking performance, compared to a target-looking strategy, even with a high tracking load [37], [38]. Moreover, this center-looking strategy does not appear to default to target-looking when the task is too difficult; rather, it may reflect a different cognitive process. Furthermore, when attention was manipulated by both a goal-driven approach and a stimulus-driven approach, participants continued to look at the center of the group of targets, rather than towards the individual targets; this suggests that center-looking reflects grouping rather than separate attentional foci [39].

Collectively, these findings suggest the pervasive existence of configuration effects in information processing. Spatial configuration may also affect the multiple identities maintenance process when multiple objects are tracked. However, these findings have been obtained either in static scenes or in standard MOT without identities. Whereas the spatiotemporal information remains the same in a static scene, it changes continuously during multiple identity tracking. Furthermore, featural properties are not involved in standard MOT. Consequently, it remains unclear whether these results can be extended to more realistic dynamic scenes.

## The Current Study

The current study explored the role of spatial configuration in maintaining successful multiple identity tracking by manipulating the targets’ overall spatial configuration during tracking. In the configuration constant condition, the spatial configuration of the targets remained a non-rigid virtual convex polygon during tracking. In the configuration collapsed condition, the virtual convex polygon lost coherence because a vertex of the virtual polygon crossed over an edge of the polygon during tracking. Experiments 1a, 1b, and 2 were conducted to investigate whether spatial configuration is involved in multiple identity-location bindings by comparing the identification performance between the two conditions. Based on those results, Experiment 3 investigated the distribution of attention among all targets at the time of the spatial configuration collapse by using a probe-dot detection task. To further reveal the effect of configuration collapse, Experiments 4 and 5 tested identification performance and error types among different targets. In Experiment 6, the spatial configuration composed by all objects (targets and distractors) was manipulated in order to further explore the constitution of the configuration.

## General Methods

### Ethics Statement

All participants provided written informed consent before participating in the experiments. The participants were reminded of their right to discontinue participation at any time. All procedures were approved by the Research Ethics Board of Zhejiang University.

### Stimuli and Procedures

**Figure 1:**
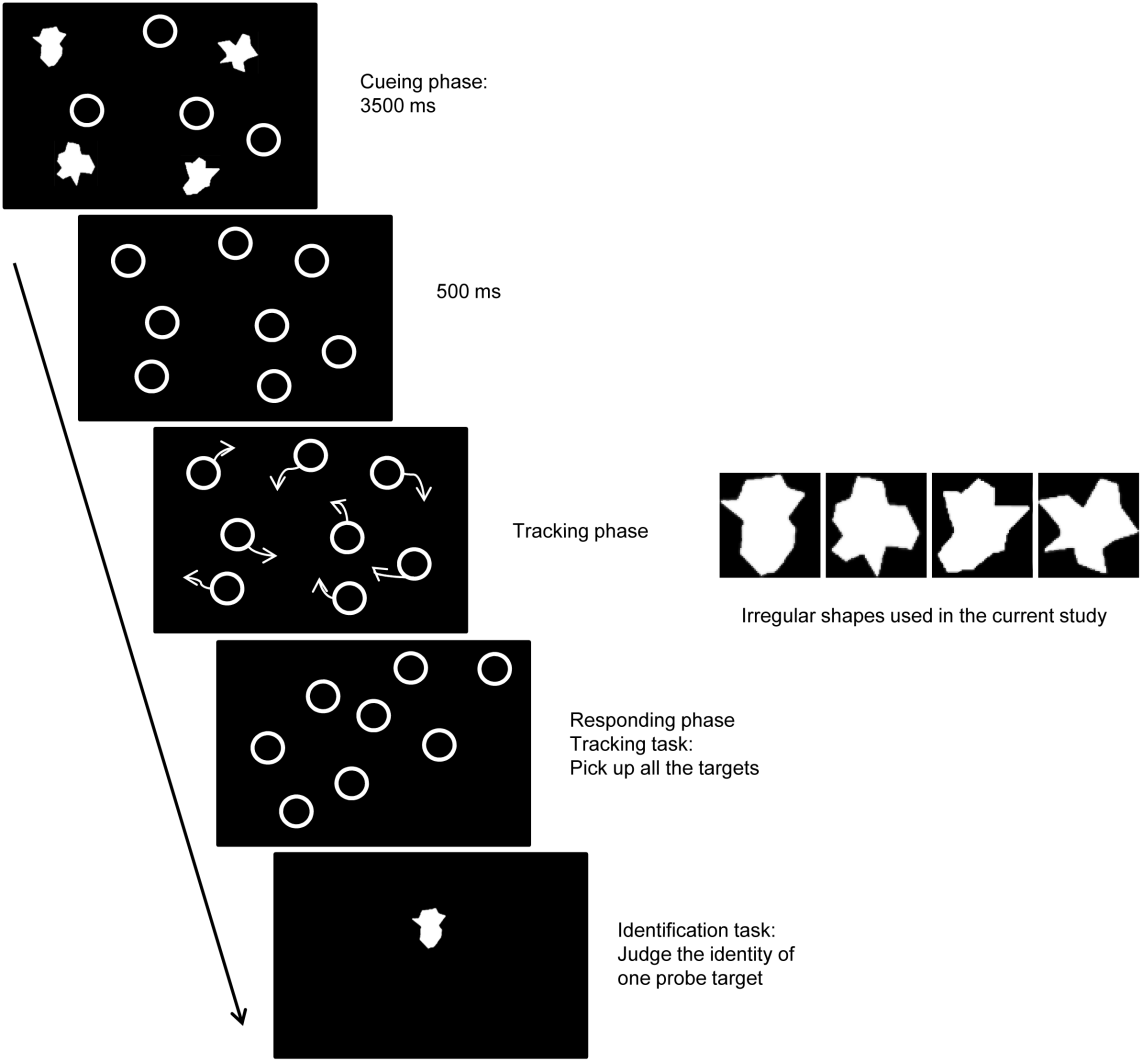
General depiction of the visual displays and stimuli. Four objects with discrete identities were used as tracking targets in the cueing phase. After cueing, the identities of the targets disappeared, and all the items were presented for 500“pick up” all of the targets and indicate whether the identity of a test target was the same as the original (see General Methods for details). The figure in the right panel illustrates the irregular shapes used as identities.

Except where noted, all six experiments used the following procedure. The experiment began with a cue phase (see Figure 1) in which four distractors and four targets with discrete identities were presented on a computer screen for 3.5 s (exceptions were Experiments 2 and 6, in which only four targets or three targets and one distractor were used, respectively). The identities were either distinct colors or distinct irregular shapes (see Figure 1, right panel). In contrast to Yantis (1992), in which the initial targets always formed a canonical polygon [13], the initial object positions in our study were generated randomly with the constraints that none overlapped and the four targets formed a virtual convex polygon. This prevented an explicit cue about grouping targets. After cueing, the identities of the targets disappeared and all of the items were presented for 500 ms, followed by a period during which all of the items started to move (the duration of movement varied across experiments). A motion algorithm was used to provide an unpredictable motion with the constraints that no object could overlap and a maximum speed of 3 pixels per frame for both the horizontal and vertical axes. This algorithm provided smooth and unpredictable movement trajectories for the objects. A set of such movement trajectories was then generated, each with 250 frames. Based on the motion pattern of the targets, these trajectories were then labeled as configuration constant or configuration collapsed trajectories. In the configuration constant condition, the spatial form of all the targets remained a non-rigid virtual convex polygon during the movement phase, but the exact form of the polygon could continue to change. In the configuration collapsed condition, the virtual convex polygon lost coherence because a vertex of the virtual polygon crossed over an edge of the polygon (except in Experiment 6; see details below). The movement of the distractors was not constrained; thus, distractors could frequently cross through the virtual polygon formed by targets in both conditions. This results in an equal average density (i.e., the distance to nearest neighbor object) in the two conditions, a factor that impacts tracking [12]. There were 48 movement trajectories in each condition. A pre-test was conducted in order to confirm that there was no detectable difference between the two conditions. In the pre-test, trials with the trajectories from each of the two conditions were intermixed, and the participants performed the identifying task. When they completed the pre-test, participants reported whether they noticed the configuration manipulation or used a configuration strategy while completing the task. None did, indicating that the two conditions were identical in appearance. Thus, it is unlikely that the participants were able to distinguish the configuration constant display from the configuration collapsed display, or that they used different strategies in each condition. The pre-test results ensure that any observed differences between the two conditions is attributable to the effect of configuration on multiple identity tracking, rather than to the participants’ use of different strategies.

Participants were instructed to make two responses on each trial after observing the motion (except in Experiment 2; see details below). First, they were to move the cursor and press the left mouse button to “pick up” (i.e., click on) all four targets (they were instructed to guess if they were unsure). Once the four targets were selected, all the items disappeared and one randomly chosen test target with identity information reappeared in its previous location. In 50% of the trials, the probe targets’ identity information was altered with the identity information of a different target. The participants then pressed a button to indicate whether the identity of the test target was the same as the original (button “J” for yes and “F” for no). The participant controlled the pace of trial presentation by pressing the spacebar to initiate each new trial. Each participant was given a 2-min practice period prior to beginning the formal experiment.

## Experiments 1a and 1b

Experiments 1a and 1b investigated whether spatial configuration affects the representation of multiple identity-location bindings during tracking. The key comparison occurred between the configuration constant and configuration collapsed conditions. In the configuration constant condition, the form of all the targets remained a non-rigid virtual convex polygon during the movement. In the configuration collapsed condition, a vertex of the virtual polygon crossed over an edge of the polygon and thus destroyed the configuration. If spatial configuration is involved in multiple identity-location bindings, then participants should show better target identification in the configuration constant than the configuration collapsed condition. Alternatively, if spatial configuration does not affect multiple identity-location bindings, then there would be no difference between the two conditions because the algorithm for these two conditions would be identical. Thus, there would be no identification performance difference between these two conditions.

### Methods

Thirty-five Zhejiang University students were paid to participate in this study (19 and 16 participants in Experiments 1a and 1b, respectively). All had normal or corrected-to-normal vision. The data from three participants in Experiment 1a and one participant in Experiment 1b were excluded due to chance-level identification performance.

Stimuli were presented on a 17-in computer monitor (100-Hz refresh rate) with a black background. The items were white circles (diameter = 1.8°, thickness = 0.15°) and the identities were either distinct irregular shapes (Experiment 1a; see Figure 1, right panel) or distinct colors (blue, red, yellow or green; Experiment 1b). The set of movement trajectories for each trial was stored offline as 250 static frames. Each frame was displayed for 20 ms for a total of 5 s of motion, and the item’s speed ranged from 0 to 10.50 deg/s. There were 96 trials (48 in each condition), and each trial had a different trajectory. Trials from each condition were intermixed randomly for each participant.

### Results and Discussion

**Figure 2:**
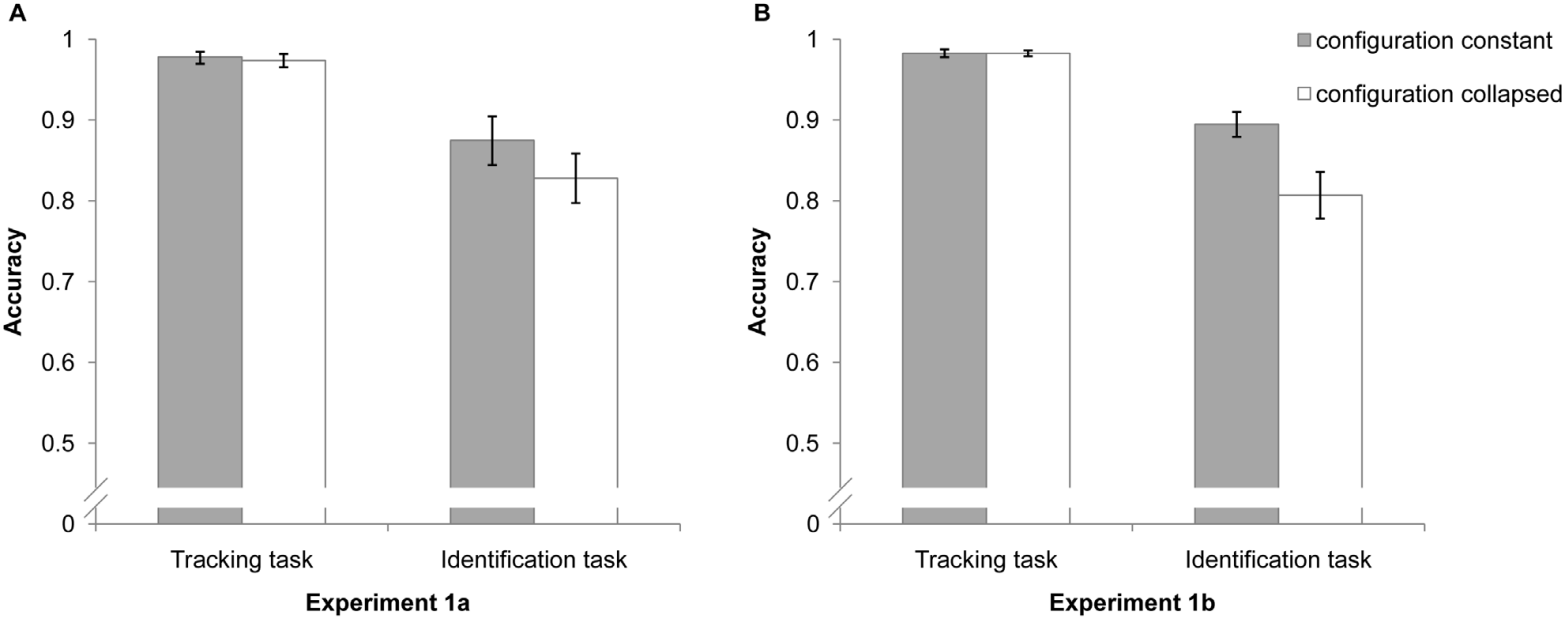
Results of Experiments 1a and 1b. A: The results of Experiment 1a. B: The results of Experiment 1b. Both panels depict mean tracking and identification performance with error bars (SE).

Tracking accuracy was defined as the percentage of correctly tracked targets averaged across trials. Identification accuracy was defined as the percentage of targets in which the identity was correctly identified. Tracking and identification accuracies from Experiment 1a are shown in Figure 2A. The overall tracking accuracy for both conditions was high (*M* = 97.8% and 97.4% for the configuration constant and configuration collapsed conditions, respectively), indicating that participants successfully tracked all four targets in both conditions and that spatial configuration collapse during tracking did not adversely affect tracking. This differs from Yantis (1992) [13], and may be due to a difference in task difficulty (i.e., our tracking task may have been easier, as evidenced by the observed ceiling performances).

The most important finding of Experiment 1a was the difference in identification performance across conditions. Only trials in which all four targets were correctly tracked were included in this analysis. Accuracy in the configuration constant condition (*M* = 87.5%) was higher than that in the configuration collapsed condition (*M* = 82.8%), *t*(15)  = 2.462, *p*<.05. Thus, the current results support the view that spatial configuration plays an important role in multiple identity tracking.

The results of Experiment 1b (see Figure 2B) were similar to those of Experiment 1a. The mean tracking accuracies were high (*M* = 98.3% for both conditions). Accuracy on the identification task was significantly better in the configuration constant condition (*M* = 89.5%) than the configuration collapsed condition (*M* = 80.7%), *t*(14)  = 3.687, *p*<.01. Note in case the current data distribution was not normal which made the current conclusion not safe, we also tested the normality of the data (using Shapiro-Wilk test) for the current experiment as well as all the following experiments. If one condition in an experiment did not meet the normality criterion, an arcsine square root transformation was applied to the whole experiment before further analysis. We found similar results, please see Table S1 in Supporting Information for details.

## Experiment 2

Experiments 1a and 1b demonstrated that spatial configuration plays an important role in the representation of multiple identity-location bindings by showing that a loss of polygon coherence impaired successful target identification. One possible explanation is that the type of configuration collapse used in those experiments reduced tracking performance [13], but this effect was not observed because participants’ performance was at ceiling. Participants may prioritize the tracking task, leaving fewer resources for the identity task when it becomes more difficult to track. Thus, the poorer identification performance observed in the configuration collapsed condition of Experiments 1a and 1b may have been caused by a trade-off between the two simultaneous tasks (tracking and identifying). To rule out this alternative explanation, in Experiment 2, the difference in tracking difficulty between the two conditions was eliminated by removing all the distractors (i.e., the participants only performed the identity task). If the decrease in target identification in the configuration collapsed condition of the previous experiments was due to tracking difficulty, then target identification accuracies should be similar for both conditions in this experiment. Alternatively, if accuracy remains poorer in the configuration collapsed condition for the identity task, the notion that spatial configuration is involved in multiple identity-location bindings will be supported.

### Methods

A group of 16 naïve participants were tested in this study. The data from one participant were excluded because of chance-level identification performance. The movement trajectories were the same as in the previous experiments, but no distractors were presented. Since there were only four targets presented on the screen, the participants only needed to perform the identity task after the tracking phase (i.e., they were not required to “pick up” the targets). All other aspects of the procedure were the same as those in Experiment 1a.

### Results and Discussion

The results of Experiment 2 were similar to those of Experiments 1a and 1b: mean accuracy was better in the configuration constant condition (89.7%) than in the configuration collapsed condition (85.7%), *t*(14)  = 2.168, *p*<.05. Together, these three experiments strongly support the hypothesis that spatial configuration plays an important role in the representation of multiple identity-location bindings.

## Experiment 3

In the previous experiments, we demonstrated that spatial configuration plays an important role in the representation of multiple identity-location bindings, namely that losing polygon coherence impaired successful maintaining of target identification. Experiment 3 further explored whether targets’ spatial configuration would guide the distribution of attention during tracking. Accordingly, we employed the probe detection task during the tracking procedure. This task has been widely used for measuring the relative distribution of attention in MOT [40]–[44].

### Methods

**Figure 3:**
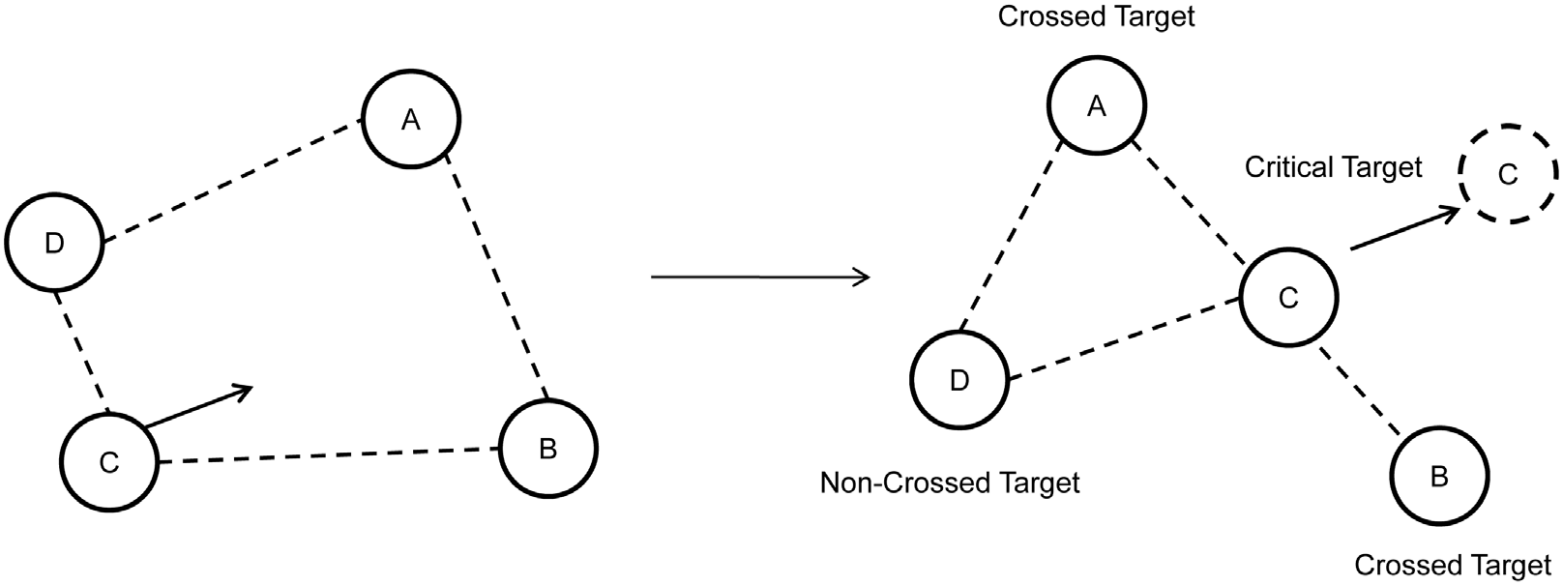
Illustration of the target categories during configuration collapse. The left panel illustrates the configuration prior to collapse, and the right panel illustrates the moment when target “C” crossed edge “AB”.

The procedure was similar to that in Experiment 1a except as noted below. Twelve naïve participants were tested. Only the configuration collapsed condition was used. The 250 static frames used in each trial were each presented for 15 ms in order to limit the duration of the experiment so as to prevent participant frustration. The probe appeared at the location of three different types of targets for 105 ms (i.e., seven frames) at the time the spatial configuration collapsed during tracking. For example, as illustrated in Figure 3, target “C” crossed over the edge of the virtual polygon (edge “AB”) and thus destroyed its coherence. Target “C” is labeled “critical target,” each vertex of the crossed edge “AB” is labeled as “crossed target” (target “A” and “B”), and the constant target (target “D”) is labeled “non-crossed target”. The probes were equally likely to appear in the location of these three target types. The probe was a gray square (0.13° in width) that appeared in the center of the target.

During the movement phase of each trial, participants were instructed to make their response as quickly as possible after detecting the probe. Key presses that occurred within 1 s of probe onset were recorded as hits, whereas those that occurred at any other time were recorded as false alarms. The participants were informed that the probe might appear at most once per trial. In addition, because the tracking performance in previous experiments was extremely high, and in order to further simplify the task, the requirement to “pick up” each target was removed. Instead, the participants were required only to perform the identification task after the reappearance of the test target at the end of tracking. The instructions stressed that participants should prioritize the identification task over monitoring for the probes.

There were 144 trials in this experiment. To prevent the participants’ predicting probe appearance, the probes only appeared in 80% of the trials. On the trials involving probe appearance, there was an 87.5% chance that the probe would appear at the moment of spatial configuration collapse (between 960 ms and 2925 ms because the motion varied from trial to trial. The right panel in Figure 3 shows the moment when target “C” crossed edge “AB”). On the remaining 12.5% of these trials, the probe was equally likely to appear at least 600 ms before or after the moment the spatial configuration collapsed.

### Results and Discussion

**Figure 4:**
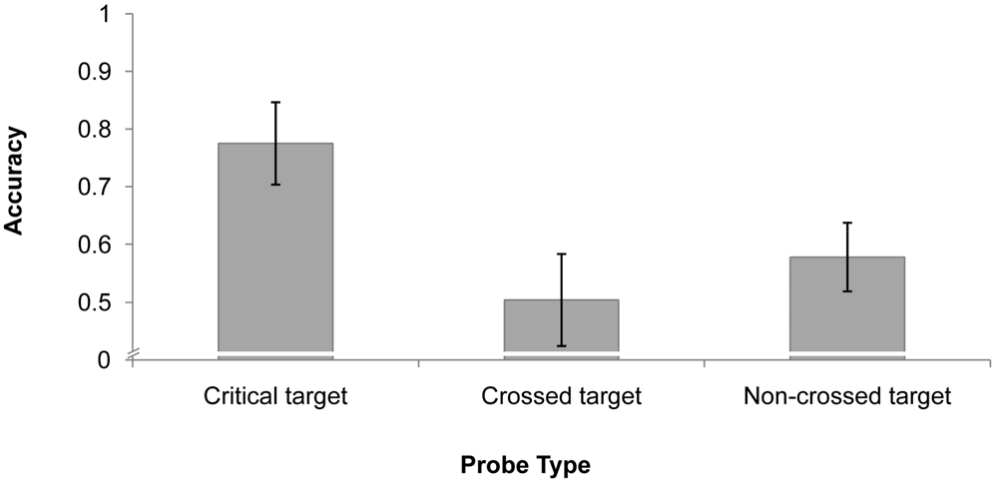
Results of Experiment 3. Mean probe performance for different targets with error bars (SE).

Participants’ mean identification performance was 77.1%. We focused on probe-detecting performance to examine the pattern of attention distribution. The probe-detecting data were analyzed only if there were no false alarms and the target was correctly identified; this ensured that the participants did perform the identification task. Detection accuracy was defined as the percentage of trials in which the probe was correctly detected. As depicted in Figure 4, a repeated-measures analysis of variance (ANOVA) indicated a significant main effect of probe type, *F*(2, 22)  = 26.551, *p*<.001. Post-hoc (Bonferroni) analysis showed that accuracy was significantly higher in the “critical target” condition (*M* = 77.5%) than the “crossed target” condition (*M* = 50.4%), *p*<.001, and the “non-crossed target” condition (*M* = 57.8%), *p*<.001, but the performance difference between the “crossed target” and “non-crossed target” conditions was not significant, *p* = .281. This suggests that the critical target (i.e., the target that violated the coherence of the spatial configuration) received more attention than the other targets.

It could be argued that the critical target may have received more attention than the other targets because it was more likely to be presented in the area that the participants tends to look. This is a possibility both because the critical target always crossed over the virtual polygon’s edge and was thus more likely to occur in the center of the screen, and because participants were more likely to be looking at the center of the display. To rule out this possibility, we analyzed only trials in which the probe was presented at least 5° from the center of the screen (63.9% of trials). The main effect of probe type was significant, *F*(2, 22)  = 18.514, *p*<.001, and accuracy in the critical target condition (*M* = 72.6%) was significantly better than that in the crossed target condition (*M* = 49.2%), *p* = .001, and non-crossed target condition (*M* = 48.9%), *p*<.001. There was no difference between the crossed target and non-crossed target conditions, *p* = 1.000.

The results revealed that at the time the spatial configuration collapsed and the virtual polygon lost its coherence, attention was more likely to be allocated to the critical target (that caused the collapse) than to the other targets. This may reflect a mechanism in which the visual system tries to maintain or rebuild the virtual polygon when it collapses in order to keep the critical identity-location binding correct.

## Experiment 4

In Experiment 3, we found that more attention was allocated to the critical target when it violated polygon coherence during multiple identity tracking than to other target types. This provides strong evidence that the critical target plays a unique role among the targets. That is, it functions as a “destroyer” in configuration collapse events. Thus, a further reasonable hypothesis is that if the visual system does form a representation of spatial configuration during multiple identity tracking, then identification accuracy may vary with the target’s role (defined by its geometric features at the time of a configuration collapse event). Experiment 4 tested this hypothesis by comparing identification accuracy for different target types.

### Methods

This procedure was similar to that of Experiment 1a except as noted below. We tested a group of 16 naïve participants. The data from two participants were excluded because of chance-level performance on the identification task. Only the configuration collapsed condition was used. The 250 static frames used in each trial were each presented for 15 ms each to control the duration of the experiment. The test target used in the identification task was not randomly chosen; rather, each of three types of target had an equal chance of being the test target. There were 144 trials, and the three conditions were intermixed randomly within participants.

### Results and Discussion

**Figure 5:**
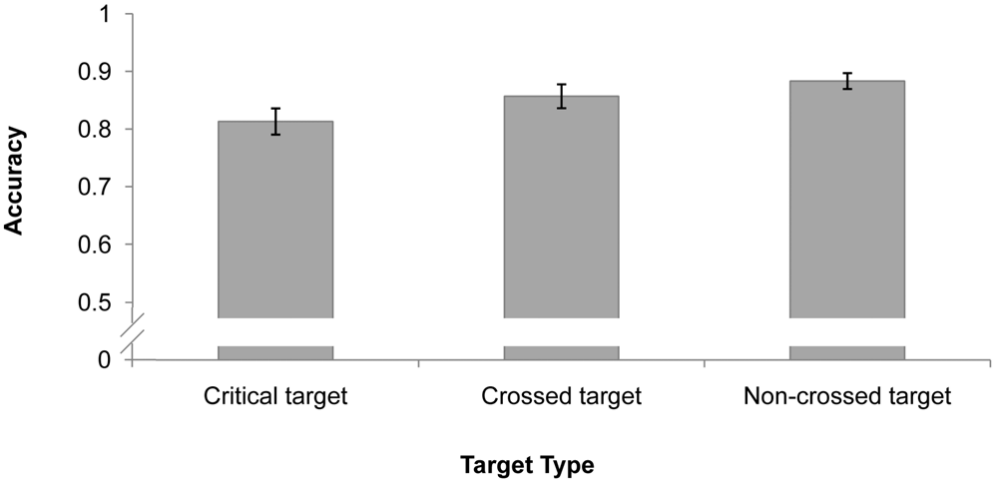
Results of Experiment 4. Mean identification performance for different target types with error bars (SE).

Overall mean tracking accuracy was high (*M* = 100.0%). The results of interest were the identification accuracy for different targets. As depicted in Figure 5, there was a significant main effect of target type, *F*(2, 26)  = 7.612, *p*<.01. Mean accuracy for the critical target (81.3%) was significantly worse than that for the crossed target (85.7%), *p*<.05, and the non-crossed target (88.3%), *p*<.05. There was no significant difference in accuracy between the crossed target and the non-crossed target conditions, *p* = .571. This result suggests that despite attention being allocated to the critical target, it is difficult to maintain and update its identity-location binding when the virtual polygon collapses.

## Experiment 5

As seen in Experiment 4, the drop in accuracy in the collapsed condition (Experiments 1a, 1b, and 2) appears to be the result of a drop in identification accuracy for the critical target. Participants were more likely to confuse the critical target with non-crossed and crossed targets. To examine this possibility, in Experiment 5, we examined the error types associated with different targets.

### Methods

The procedure was similar to that of Experiment 4 except as noted below. We tested 20 new participants. Data from two participants were excluded due to chance-level performance on the identification task. The identification task was modified such that error types associated with the test target were easily measured. In particular, rather than asking participants to judge whether the identity of the test target was the same as the original, one test target without identity was presented. The four target identities were also presented in a row on the display (in the center of the screen if the test target was not there, otherwise on the bottom of the screen). The participants were told to “pick up” the identity of this test target by pressing the keyboard buttons “1,” “2,” “3,” or “4” (for the leftmost identity to the rightmost identity, respectively).

### Results and Discussion

Tracking, identification accuracy and error types were analyzed. The overall mean tracking accuracy was high (99.0%). A similar trend was found as in Experiment 4; there was a significant main effect for target type, *F*(2, 34)  = 3.908, *p*<.05. Accuracy of the critical target identification (*M* = 75.3%) was significantly worse than that of the non-crossed target (*M* = 80.8%), *p*<.05, the accuracy difference between the critical target and the crossed target (*M* = 78.2%) was not significant, *p* = .555, and the accuracy difference between the crossed target and the non-crossed target was not significant, *p* = .488.

The results of interest were the identification error types for different targets. Since there were one critical target, two crossed targets and one non-crossed target, the probability of the critical target being misidentified as the identity of the crossed target or the non-crossed target was 2/3 and 1/3, respectively. The probability of the non-crossed target being misidentified as the critical target or the crossed target was 1/3 and 2/3, respectively. The probability of the crossed target being misidentified as the critical target, non-crossed target or the other crossed target was equal (1/3). Considering the relatively high identification accuracy (e.g., several participants made fewer than five mistakes for certain test targets), there were not sufficient data to analyze each individual participant, so all participants’ data were combined. There were 205 misidentifications of critical targets; 74.1% (152) were associated with the crossed target’s identity. Participants were more likely to confuse the critical target with the crossed targets (compare to the 2/3 probability), χ^2^_(1)_ = 5.161, *p*<.05. The percentage of non-crossed targets misidentified as a crossed target was 63.5% (101 of 159), which was not different from the 2/3 probability, χ^2^_(1)_ = 0.708, *p* = .400. The percentages of crossed targets misidentified as critical targets, non-crossed targets and the other crossed target were 45.5%, 26.4%, and 28.1% respectively (81, 47, 50 for each), and were significantly different from theoretical probabilities, χ^2^_(2)_ = 11.944, *p*<.01, crossed targets were more likely to be misidentified as critical targets.

This result suggests that a participant was more likely to confuse the critical target with the crossed targets when the configuration collapsed. Considering that the critical target crossed the edge of the virtual polygon composed by the crossed targets, the observed confusion may reflect the interruption of the updating of the identity-location bindings process. If multiple bindings were updated in turn based on the virtual polygon, one crossed target would always update after the other when the configuration remained. Once the critical target crossed the edge and destroyed the configuration, it might have caused the participants to falsely bind the crossed target identity to the critical target, and vice versa. The non-crossed target, however, suffered less from the collapse, and thus made equal misidentifications.

## Experiment 6

The movement of the distractors was not constrained in Experiments 1a and 1b, such that the distractors frequently crossed through the virtual polygon formed by the targets. However, identification performance still significantly differed between the configuration constant and configuration collapsed conditions. This indicates that the distractors may not affect the construction of spatial configuration and is consistent with previous findings that distractors were inhibited during MOT [43], [45]. However, there is also evidence suggesting that distractors could be encoded under certain conditions [46]–[49]. It is still possible that the distractors are integrated into the construction of spatial configurations, especially when it is easy to form the targets and distractors into a virtual polygon. Experiment 6 investigated this possibility. Since it is harder to construct and maintain a pentagon, and to maximize the chance that the distractors were involved in constructing the virtual polygon, only three targets and one distractor were used. In the configuration constant condition, these four objects always formed a convex polygon.

### Methods

The procedure was similar to Experiment 1a except as noted. There were 12 naïve participants. The initial position of the four objects formed a convex polygon. During the tracking phase, the three targets were constrained as a triangle, and the movement of the distractor was manipulated in two different ways. In the configuration constant condition, the distractor never crossed the triangle and the form of all four objects remained a convex polygon; in the configuration collapsed condition, the distractor crossed over the triangle, thereby violating the convexity of the form of the four objects. There were 120 trials: each condition contained 60 trials, and trials from the two conditions were intermixed randomly for each participant. The 250 static trajectory frames were each presented for 10 ms in order to increase task difficulty (because the participants tracked three targets).

### Results and Discussion

**Figure 6:**
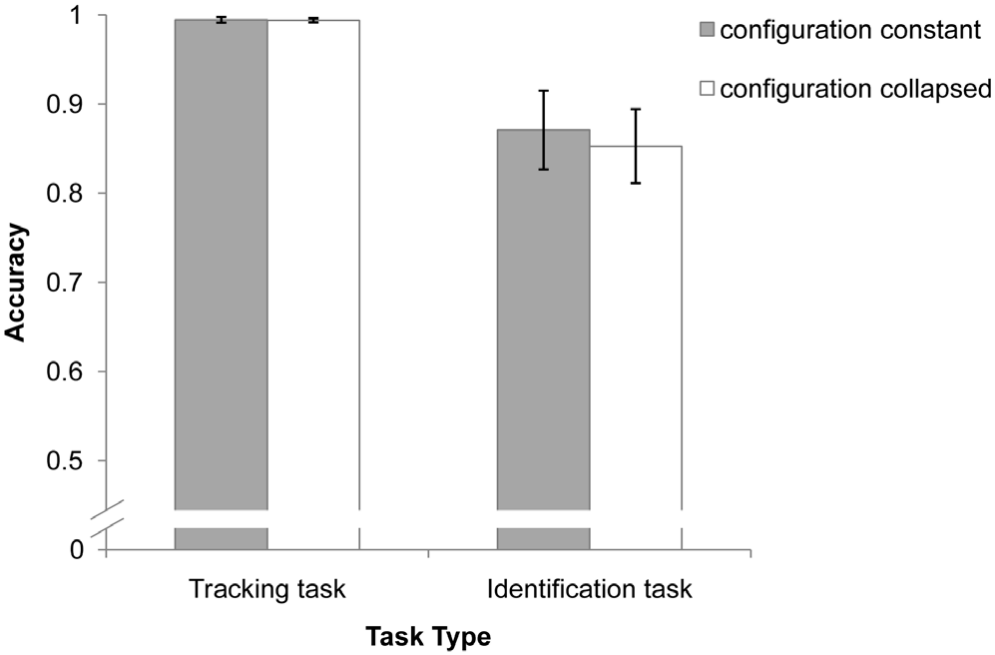
Results of Experiment 6. Mean tracking and identification performance with error bars (SE).

Tracking and identification accuracy were analyzed. As shown in Figure 6, the overall mean tracking accuracies were high for both conditions (99.4%). Identification accuracy was analyzed only if all three targets were correctly tracked. There was no significant difference in identification accuracy between the configuration constant (*M* = 87.1%) and configuration collapsed conditions (*M* = 85.3%), *t*(11) = 1.096, *p* = .296), indicating that regardless of whether the distractor violated the configuration, performance on the identification task was not affected. In other words, the spatial configuration of multiple identity-location bindings is not affected by the distractor, and the visual system constructs the virtual polygon only by using the targets.

## General Discussion

In the current study, we investigated how multiple identity-location bindings are organized when observers track multiple identity targets in dynamic environments. These experiments revealed three basic findings. First, spatial configuration plays an important role in multiple identity tracking. When the coherence of the virtual polygon formed by the targets was destroyed, observers’ identification accuracy was significantly impaired (Experiments 1a, 1b, and 2). Second, destruction of the spatial configuration affected dynamic attention allocation and the identification of different targets (Experiments 3–5). The visual system allocated more attention to the critical target when it violated the coherence of the spatial configuration. Nonetheless, the observers’ identification accuracy for that critical target was worse than that for the other targets, and critical targets were more likely to be confused with crossed targets. Third, the distractors were not integrated into the construction of spatial configuration, since observers’ identification performance was not affected whether or not the distractor violated the configuration (Experiment 6).

There are several reasons why we believe that spatial configuration is not simply a high-level strategy that assists in performing the task, but more likely an implicit perception process involved in multiple identity tracking. First, the only constraint on the target’s initial position and motion in the configuration constant condition was to maintain a virtual convex polygon. The exact form of the polygon continued to change during the movement phase. The algorithm used to generate the trajectories for both conditions was identical, and we mixed these two conditions during the experiments. Thus, it was difficult for participants to detect that there were two different configuration conditions (moreover, the pre-test data indicated that none of the participants detected the difference in conditions). Second, we tested naïve participants without knowledge of the motion pattern (constrains on how targets move) or the purpose of the study. From the participants’ perspective, they simply tracked several randomly moving unique targets. Third, there were no explicit instructions directing participants to form the targets into a virtual polygon. Fourth, none of the participants reported that they had noticed the spatial configuration of the targets. Finally, our major finding was the impairment of identification performance, which is unlikely to be the result of participants’ intentional strategies, because they were encouraged to perform as well as possible while maintaining the identity-location bindings of the moving objects. Collectively, these results indicate that participants were unlikely to engage in a high-level strategy, unless spatial configuration was an implicit process. Thus, we are confident in asserting that this is not a strategy.

## Spatial Configuration in Multiple Identity Tracking

Our observation that destroying the coherence of the virtual polygon affects identification performance strongly supports the hypothesis that spatial configuration plays an important role in representing multiple identity-location bindings. Combining previous findings [13]–[15], [18], [19] with those from the current study, we suggest that there is a configuration-based updating process in which the representation of spatial configuration may serve as an index for updating multiple identity-location bindings during tracking. While tracking multiple unique objects in a dynamic environment, observers may mentally construct a virtual polygon with the tracking targets as the vertices. Observers may then attach individual identities to the vertices of the polygon and update them in the order of adjacent vertices (i.e., clockwise or counter-clockwise) during tracking. When the coherence of the polygon is destroyed, the updating order of identity-location bindings will be interrupted, resulting in impairment of the identification performance (as shown in Experiments 1a, 1b, and 2). Maybe in order to rebuild the configuration, additional attentional resources are allocated to the critical target, which serves as the “destructor” of the configuration and therefore becomes the most important object in the rebuilding process. This leads to the changes in the attentional distribution that were exhibited in Experiment 3. Since the additional attention is used to rebuild the configuration rather than to maintain the identity-location binding of the critical target, and the updating sequence is interrupted at the critical target, it is not surprising that its identity binding fails (Experiment 4). The finding that the critical target was more likely to be mis-bound with crossed targets’ identities (Experiment 5) provides additional evidence for our configuration-based updating explanation. According to this explanation, the identity-location bindings of the two crossed targets (e.g., in Figure 3 left panel, Targets “A” and “B”) will be updated one after the other until the configuration is destroyed. When the critical target crosses the edge “AB,” it is inserted into the updating sequence between two crossed targets. Thus, the identity mis-binding usually occurs between the critical and crossed targets (see Figure 3, right panel). However, it is worth noting that the conclusion of Experiment 5, to some extent, suffered from the deficient analyzing method, since we pooled all participants’ data (because there were only very few error trials in certain conditions for many participants). Future studies are required to verify the conclusion of this experiment. Taken together, our findings imply the existence of a configuration-based updating process in multiple identity tracking. Further research may be required to verify the current explanation (e.g., using eye-tracking equipment to test the relationship between eye fixation and identification performance of different target types).

Our study sheds important light on current models of MOT. First, the grouping model [13] suggests that all of the targets are grouped into one higher order object with each target as a vertex in a virtual polygon. Our study provides further evidence regarding the importance of spatial configuration, which strongly affects successfully maintaining identity tracking as wells as MOT. Second, the visual indexes (FINSTs) or object files model (for reviews, see [50], [51]), which proposes that multiple tokens (preattention index or object file) keep track of multiple targets and their identities, may need some refinement. The current results suggest that these multiple tokens may not be represented separately, but rather integrated according to their spatial information. Of course, it is still possible that FINSTs are holding on to the individual vertices of the polygons (in order to keep track of that shape). However, a higher-order structure describing the organization of these FINSTs may be necessary to accomplish multiple identity tracking. The models that propose rapid-switching [2], [50] among targets may also need to take the spatial configuration into account.

Our study also provides a new perspective from which to view previous findings. A handful of studies have found that observers are poor at identifying targets even when they have been tracked; these results implied that the targets’ identities may not be encoded or can be easily switched between targets [14], [15]. In contrast, other studies have found that featural information is accessible during tracking, and that observers can hold approximately two targets in this type of task [16], [17]. These are seemingly contradictory findings, but those studies did not control the spatial configuration of the targets. Identification accuracy may decrease as configuration collapse increases; however, tracking accuracy could be more robust because changing the relative sequence of targets has no influence on tracking (as shown in Experiments 1a and 1b). As observers were asked to label all the identities of tracked targets (see Pylyshyn [14]), this may result in acting as though identities were switching between targets.

## Spatial Configuration and Capacity Limitation

The capacity limitation of multiple identity tracking has been actively investigated in the literature. It has been shown that identification capacity is significantly influenced by set size, target speed and duration of the tracking phase [14], [17], [19]. Our results suggest that the pattern of the targets’ motion may be another influence on identity. Multiple identities are more easily updated and maintained if the coherence of spatial configuration remains unchanged. For typical dynamic identity tracking, if the target set size is larger than four, it is difficult to construct a virtual convex polygon when tracking begins because the targets’ position is random. Even if a configuration is constructed, it will frequently collapse due to the uncontrolled motion of the targets. In this case, it is nearly impossible to maintain a virtual polygon that retains its coherence when all the targets move randomly; identification performance decreases accordingly. If the set size is small, such as two or three, it is easy to construct and maintain a triangle or line in most situations; the coherence of the triangle (or line) is rarely destroyed. Thus, high identification accuracy will be reached in these situations. When target size increases to four, it may be possible to construct a virtual convex polygon, but the configuration may collapse in conditions in which all the targets move randomly without any constraints; this results in varied identification performance, as observed in the current study.

The other factors that influence the identification capacity (e.g., target speed, tracking duration) can also be explained by the spatial configuration. As the targets’ speed or tracking duration increases, the motion trajectories of the targets increase in distance and complexity, causing more configuration collapses and impaired identification performance. This may also explain why the identification capacity limitation in our studies (about 3.2 for the configuration constant condition and 2.7 for the configuration collapsed condition) was somewhat higher than in previous findings (about 2 items; e.g., see [16], [17], [19]). In previous studies, targets’ initial position and motion were usually uncontrolled, and so participants may not have been able to construct the virtual polygon at the beginning of tracking. Even for those who do construct it, the spatial configuration will more frequently collapse as a result of the uncontrolled motion.

The tracking capacity limitation may also be significantly influenced by the spatial configuration. For example, Yantis (1992) demonstrated that tracking performance could be improved under the convexity and rigidity constraints of targets’ configuration [13]. The tracking process could be more robust than the identity process because changing the relative sequence of targets does not influence tracking when the configuration collapses. One may still benefit from the configuration if all targets are well organized (e.g., they form a convex polygon). Moreover, recovering a lost target may also be easier, especially when the set size is large.

Recently, Hudson and colleagues [52] proposed a two-stage model of multiple identity tracking, involving a tracking stage that first segregates the targets from the distractors and an identification second stage that associates a unique identity with each target. We suggest that spatial configuration may play distinct roles in these two stages. In the tracking stage, spatial configuration serves as a way to distinguish targets from distractors and a method of target recovery if a target is lost. In this stage, targets could easily be “picked up” and maintained if they are well organized, and if they are lost during tracking, the visual system could use the form information to re-track that target. In the identification stage, the relative sequence of targets is crucial for maintaining and updating multiple identity-location bindings. Spatial configuration is more likely to serve as a sequence index for the updating process. When the configuration collapses, the relative sequence of targets is changed. This affects the updating process and results in poorer identification performance, especially for the critical target. The reconstruction of the virtual polygon is also more difficult at this stage compared to the tracking stage.

## Conclusion

By manipulating targets’ motion pattern during dynamic tracking, our study showed that spatial configuration plays an important role in multiple identity tracking. Multiple identity-location bindings may be maintained and updated by a configuration-based updating process in which the configuration serves as the index for the updating. These results also add to the field of dynamic allocation of visual attention and perceptual organization.

## Supporting Information

Table S1 **The Test of Normality and Statistical results after transformation.**
(DOCX)
