# Supplementary material for: The Role of Spatial Configuration in Multiple Identity Tracking
Source: PLoS One. 2014 Apr 9;9(4):e93835. doi: 10.1371/journal.pone.0093835 (PMC3981724; doi:10.1371/journal.pone.0093835)
Supplement: Table S1 — The Test of Normality and Statistical results after transformation. (DOCX) [file pone.0093835.s001.docx]

# Supporting Information

To reach a solid conclusion, we also examined the normality of data of each condition using Shapiro-Wilk test. If one condition in an experiment did not meet the normality criterion, an arcsine square root transformation was applied to the whole experiment before further analysis (see Table S1).

In Experiment 1a, the constant condition did not meet the normality criterion. After data transformation we found the result reached significance (*p* = .06).

In Experiment 1b, both conditions met the normality criterion.

In Experiment 2, both conditions did not meet the normality criterion. After data transformation we found the result reached marginal significance.

In Experiment 3, the critical-probe condition failed the normality criterion. After data transformation we found the similar results as before.

In both Experiments 4 and 5, all conditions met the normality criterion.
 In Experiment 6, both conditions did not meet the normality criterion. However, after data transformation the result kept the same as before the transformation.

To sum up, albeit there were certain conditions not having a normal distribution, they did not change our conclusion. The conclusions of the current study are safe.

**Table S1** The Test of Normality and Statistical results after transformation

|  | Data normality testing via Shapiro-Wilk  for each condition | | | | Results after arcsine square root transformation |
| --- | --- | --- | --- | --- | --- |
| Exp.1a | Constant: *p* <. 01 | | Collapsed: *p* > .05 | | *t*(15) = 3.036, *p* < .01 |
| Exp. 1b | Constant: *p* > .10 | | Collapsed: *p* > .05 | | *Not applied* |
| Exp. 2 | Constant: *P* < .01 | | Collapsed: *P* < .01 | | *t*(14) = 2.029, *p* = .06 |
| Exp. 3 | Critical probe:  *p* < .05 | Non-Crossed:  *p* > .10 | | Crossed:  *p* > .10 | *F*(2, 22) = 35.367, *p* < .001  Critical Vs. Crossed: *p* < .001  Critical Vs. Non-crossed: *p* < .001  Crossed Vs. Non-crossed: *p* = .32 |
| Exp. 4 | Critical target:  *p* > .10 | Non-Crossed:  *p* > .10 | | Crossed:  *p* > .10 | *Not applied* |
| Exp. 5 | Critical target:  *p* > .05 | Non-Crossed:  *p* > .10 | | Crossed:  *p* > .10 | *Not applied* |
| Exp. 6 | Constant: *p* < .01 | | Collapsed: *p* < .05 | | *t*(11) = 1.871, *p* = .09 |
